# Physical activity and constipation: A systematic review of cohort studies

**DOI:** 10.7189/jogh.14.04197

**Published:** 2024-11-22

**Authors:** Jiahe Cui, Fangfang Xie, Hongyu Yue, Chaoqun Xie, Jianwen Ma, Haotian Han, Min Fang, Fei Yao

**Affiliations:** 1 Shanghai municipal Hospital of Traditional Chinese Medicine, Shanghai University of Traditional Chinese Medicine, Shanghai, China; 2 School of Acupuncture-Moxibustion and Tuina, Shanghai University of Traditional Chinese Medicine, Shanghai, China; 3 Shuguang Hospital, Shanghai University of Traditional Chinese Medicine, Shanghai, China

## Abstract

**Background:**

Constipation significantly impacts quality of life and is a common public health issue. For affected individuals, especially those who are inactive and experience constipation symptoms, it is recommended to engage in physical activity (PA) to improve their condition. However, the relationship between PA and improvement in constipation remains unclear. We performed this systematic review of cohort studies to evaluate this potential association.

**Methods:**

We systematically searched the Embase, Cochrane Library, PubMed, and CINAHL databases for all cohort studies examining the relationship between PA and constipation from the inception of the databases up to 5 November 2023. We calculated the reported risk ratios (RRs) and 95% confidence intervals (CIs), conducted a random effects model, and performed a subgroup analysis based on factors such as gender, geographic region, and PA intensity to comprehensively explore the link between PA and constipation. Furthermore, we used the Newcastle-Ottawa Scale to evaluate the quality of the studies included in our analysis.

**Results:**

The analysis included 13 studies with 119 426 participants and 63 713 cases. The results indicated that higher levels of PA were associated with a decreased risk of constipation compared with lower levels of PA (RR = 0.69; 95% CI = 0.88–0.83) and moderate levels of PA (RR = 0.87; 95% CI = 0.79–0.95). Furthermore, adherence to international PA guidelines was correlated with a significantly reduced risk of constipation (RR = 0.87; 95% CI = 0.81–0.93). Notably, the risk of constipation was lowered among Asian populations (RR = 0.67; 95% CI = 0.56–0.79) and Oceanian populations (RR = 0.72; 95% CI = 0.63–0.83) who engaged in regular PA. Moreover, when comparing the risk of constipation between men and women collectively, PA was associated with a 34% lower risk (RR = 0.66; 95% CI = 0.55–0.80).

**Conclusions:**

The study findings indicated that moderate to high levels of PA significantly reduced the risk of constipation, showing a negative correlation between PA and constipation.

**Registration:**

PROSPERO: CRD42023479653.

Constipation is typically caused by the dysfunction of the colon or rectum, pelvic floor, and anus, leading to difficult defecation, incomplete defecation volume, and less than three bowel movements per week. Patients may also experience other symptoms such as abdominal discomfort, abdominal distension, straining during defecation, and anorectal obstruction [1]. The global prevalence of functional constipation that meets the diagnostic requirements of the Rome Standard IV is 10.1%, with varying incidence rates across countries [2]. Although constipation is not directly life-threatening, it may increase the risk of some future clinical outcomes. For example, compared with non-constipated patients, constipated patients have a higher cumulative incidence of all-cause mortality, as well as a higher crude cumulative rate of coronary heart disease and ischemic stroke [3]. Constipation status and severity are also associated with a higher risk of incident chronic kidney disease and end-stage renal disease [4]. Moreover, as the duration of constipation increases, the likelihood of developing colorectal cancer and benign colorectal tumours rises among affected individuals [5].

Constipation is often related to a range of complications, and untreated constipation can potentially progress to more serious conditions such as faecal impaction or intestinal perforation. These developments significantly reduce the quality of life for affected individuals and impose a substantial economic burden. Consequently, timely intervention is crucial to mitigate the adverse consequences associated with constipation [6]. Several studies have indicated that women and older individuals are primary risk factors for increased constipation occurrence [7–9].

Constipation is primarily treated through medication, typically administered gradually based on the patient’s condition. Some common examples include bulk-forming laxatives, osmotic laxatives, stimulant laxatives, prosecretory agents, and prokinetic agents [10]. However, medication use may lead to side effects such as abdominal bloating, diarrhoea, abdominal pain, nausea, vomiting, headache, and electrolyte imbalance [11]. While non-pharmacological therapies, such as lifestyle and diet adjustments, biofeedback techniques, and neurostimulation, are also used to treat the condition, they have limitations or require further research to confirm clinical efficacy [12]. Thus, it is crucial to enhance current interventions or establish their clinical benefits in constipation treatment, while new techniques are being developed.

Nonpharmacological treatment, however, is the initial step in treating functional constipation [13], with regular physical activity (PA) being key in this sense. The World Health Organization (WHO) defines PA as ‘any physical movement produced by the skeletal muscles that consumes energy’ [14]. As such, it has multiple benefits for the body, contributing to the primary prevention of 35 chronic diseases and offering a higher therapeutic index compared to medication [14]. As for constipation, a lack of PA may increase the length of constipation episodes, while engaging in PA can prevent and delay the onset of diseases [15]. Studies have shown that PA intensity has a dose-effect relationship with constipation [16], the mechanism of which remains unclear and may be related to promoting colon movement and reducing intestinal blood flow [17]. Currently, there is limited randomised controlled research regarding PA intervention in constipation, and the results from different studies have varied. Therefore, the relationship between PA and constipation remains unclear [18,19].

PA offers advantages such as being free of side effects, sustainable, and cost-effective as a non-pharmacological treatment for constipation. Currently, few meta-analyses have been performed to evaluate the effects of PA on constipation. Therefore, we conduct a systematic review to address this issue. Specifically, we assessed whether PA can reduce constipation risk, the effect of different PA levels on constipation, and the relationship between several important potential modifiers for constipation, including age, sex, body mass index (BMI), smoking, alcohol consumption, and fibre intake. The findings from our research could offer insights and quantifiable data on the association between PA and constipation risk.

## METHODS

We followed the PRISMA guidelines in reporting the findings from this systematic review and registered it within PROSPERO (CRD42023479653).

### Literature search

We evaluated relevant human-centred, English-language studies available in Embase, Cochrane Library, PubMed, and CINAHL, and published from the inception of each database to 5 November 2023. We queried these databases by connecting descriptors of PA using the Boolean operator ‘OR’, after which we combined that query with another that used terms related to constipation outcomes using the term ‘AND’ (**Online Supplementary Document**). We based our search strategy on human-centred English research articles and reviewed relevant reference lists to identify additional studies that aligned with our search criteria.

### Inclusion and exclusion criteria

Two authors (JHC and FFX) independently reviewed the titles and abstracts, followed by the full text of retrieved studies for eligibility. To be included, studies had to have investigated the relationship between PA and constipation; used an observational cohort design; and reported odds ratios, risk ratios (RRs), or hazard ratios with their corresponding 95% confidence intervals (CIs) or sufficient information to calculate these indices. We excluded studies whose full text could not be located, those that did not report or have relevant data available; those without designs of interest (editorial, review, meta-analysis, comment, new, letter, practice guideline); those that combined PA with diet, medication, or other interventions; duplicate reports; and those covering unrelated topics. In cases where there was a discrepancy between the two reviewers regarding the inclusion of literature, a third experienced researcher (FY) was invited for discussion until a consensus was reached.

### Data extraction

To investigate the potential association between PA and constipation, we extracted key information from the articles into a pre-designed extraction table. These data included the first author’s surname, number of cases, date of publication, gender distribution, sample size, research design and location, and the classification of PA into three levels. We also extracted RR estimates, their, 95% CIs, and adjustment factors in total and separately for men and women. When different types of PA were reported and evaluated in the articles, special attention was given to vigorous exercise, quantitative PA, and lifelong PA. The evaluation of the quantitative PA involved assessing the frequency of PA, or more specifically, the number of PA cycles per week.

For studies which used high PA levels as the reference group for the RR assessments, we converted the reported RR assessments to reciprocal values.

### Study quality

Two assessors (JHC and FFX) independently used the Newcastle-Ottawa Scale to evaluate the quality of the studies [20]. In case of disagreement, a third experienced researcher (MF) was invited to decide on the final evaluation. The scale has a total score of nine and can assess cohort studies (i.e. selection, comparability, and outcome) and case-control studies (i.e. exposure, selection, and comparability). Studies with a score of four were considered low quality, while those with a score of five were considered high quality.

### Data analysis

We measured the association between PA and constipation risk using the RR estimate value explained by the odds and risk ratio. Adjusted RRs reported in the original literature were used when available for the RR value. Otherwise, we used Stata, version 11.0 (StataCorp, College Station, Texas, USA) to calculate the RRs and their 95% CIs. The calculation of the natural logarithm of the RR estimate and its corresponding standard error (SEsi) was computed as follows:

SE*si* = *(log(upper 95% CI limit of the RR) − log(RR))/1.96*.

We used a random-effects model to interpret the weighted average of the logarithm of RRi. Furthermore, the logarithm of RRi was weighted by dividing by *wi = 1 / (si*^2^ *+ t*^2^*)*, where *si* represents the standard error of the logarithm of RR, and *t*^2^ denotes the maximum likelihood estimate of the total variance.

Heterogeneity testing was conducted using Cochran’s Q and the *I*^2^ statistic [21]. An *I*^2^<25% is considered an important indicator of heterogeneity. Moreover, we assessed for publication bias by using funnel plots, a graphical display of the sample size plotted against the effect size for the studies included in a meta-analysis, widely used to examine bias in the results of meta-analyses [22,23]. We also used Begg’s rank method [24] and Egger’s linear regression method [25] for this purpose, where a *P*-value <0.05 was deemed statistically significant.

We conducted a subgroup analysis to examine the association between PA and constipation risk by gender (male, female), geographic region (Asia, Europe, North America, Oceania), PA intensity (high, medium, low), adjusted BMI (yes, no), adjusted sex (yes, no), adjusted age (yes, no), adjusted alcohol consumption (yes, no), adjusted smoking (yes, no), and adjusted fibre intake (yes, no). This comprehensive analysis allowed us to investigate the specific relationships between these variables and their impact on the constipation risk.

We conducted the statistical analysis in Stata, version 11.0. (StataCorp, College Station, Texas, USA). The RR values were reported alongside their 95% Cis, and a significance level of 0.05 was considered to indicate a statistically significant difference.

## RESULTS

### Study characteristics

Our initial search identified 4968 studies. After removing 740 duplicate studies, we excluded 4120 studies based on title and abstract screening, resulting in 108 remaining studies. Of these, 92 were excluded due to an inability to extract RR data, and three were non-English language studies. Finally, we included 13 studies [26–38], yielding 15 different estimates of the RR (Figure S1 in the **Online Supplementary Document**) for 119 426 participants in the meta-analysis, 63 713 of whom were cases.

The 13 cohort studies were conducted in five regions: six in Asia, four in Europe, two in North America, and one in Oceania (Table S1 in the **Online Supplementary Document**). Three studies enrolled males only, five only females, and the rest enrolled both males and females. The degree of covariate adjustment varied, and the risk estimates of nine studies were adjusted for factors such as age, smoking, alcohol consumption, and BMI. Three studies were adjusted for fibre intake and water intake. Based on the six studies we included [26,27,33–35,37], the PA levels were defined as high PA (>150-minute/week), moderate PA (30–150-minute/week), and low PA (<30-minute/week).

### Subgroup analyses

#### Comparison of high and low PA levels

The findings of the random-effects model showed that high PA levels were associated with a reduction in constipation risk when compared with low PA levels (RR = 0.69; 95% CI = 0.58–0.83) (Figure S2 in the **Online Supplementary Document**).

#### Gender

We conducted separate analyses based on gender, which showed that PA was associated with an increased constipation risk for males (RR = 0.57; 95% CI = 0.27–1.23) and females (RR = 0.77; 95% CI = 0.54–1.10). However, when comparing males and females together, PA was associated with a reduction in the constipation risk (RR = 0.66; 95% CI = 0.55–0.80) (Figure S3 in the **Online Supplementary Document**).

#### Geographical regions

Our analysis of various geographic regions showed that PA lowered the constipation risk in Asian populations (RR = 0.67, 95% CI = 0.56–0.79) and Oceanian populations (RR = 0.72, 95% CI = 0.63–0.83) (Figure S4 in the **Online Supplementary Document**).

#### Publication bias

Based on our analysis of funnel plots, Begg’s rank method, and Egger’s linear regression method, we found no evidence of publication bias among the included studies (*P* > 0.05) (Figures S5–7 in the **Online Supplementary Document**).

#### Constipation incidence by PA level

We conducted meta-analyses to compare the risk estimates of moderate and high levels of PA from 10 studies and low levels of PA from eight studies in relation to constipation risk. The results showed that moderate PA levels reduced the constipation risk compared with low levels of PA (RR = 0.85; 95%CI = 0.72–1.00, and high PA levels also reduced the risk of constipation compared with medium PA levels (RR = 0.87; 95%CI = 0.79–0.95). Furthermore, our current evidence demonstrated that adhering to the international PA guidelines can effectively reduce the risk of developing constipation (RR = 0.87; 95% CI = 0.81–0.93) (Figures S8–10 in the **Online Supplementary Document**).

#### Covariate analysis

We also investigated the influence of confounding factors such as smoking (three studies), alcohol consumption (three studies), age (seven studies), gender (three studies), BMI (five studies), and fibre intake (four studies) in each study. All studies indicated that PA increased the constipation risk, but this was not statistically significant.

#### Study quality

In terms of quality, the studies had a mean score of 6.23 (standard deviation = 1.12) and a median of 6 (interquartile range = 5–7) (Table S2 in the **Online Supplementary Document**). The studies we included covered various confounding factors, such as smoking, alcohol consumption, age, gender, BMI, and fibre intake. All studies showed improvements in constipation symptoms. The quality assessment of the included studies was consistently above five, indicating high research quality. In previous research [39,40], we found that if the quality of the studies included in the literature was higher, publication bias was lower, and the results were more stable.

## DISCUSSION

### Association between PA and constipation risk

In general, PA has been shown to have a beneficial effect on health across race, ethnicity, gender, and age groups, as well as to promote normal growth and development, improves body sensations, functions, and sleep status, and reduces the risk of chronic diseases [41]. Conversely, inactivity may increase the risk of chronic diseases and conditions [41]. Despite this documented dose-response relationship between PA intensity and certain health outcomes, health gains may not consistently correlate with PA intensity. Moreover, some health benefits may still occur even at PA levels below recommended thresholds [42,43]. For optimal health, adults should engage in more than 150 minutes of moderate-intensity PA or more than 75 minutes of vigorous-intensity PA weekly, while children and adolescents require an average of 60 minutes of moderate-to-vigorous PA per day [14]. Therefore, we conducted a random-effects meta-analysis that examined the relationship between PA and constipation across different PA levels (high vs low PA, high vs moderate PA, and moderate vs low PA), as well as on meeting vs not meeting the international PA guidelines. The findings showed that PA serves as a protective factor against constipation. When compared with low PA, both moderate PA (RR = 0.85; 95% CI = 0.72–1.00) and high PA (RR = 0.69; 95% CI = 0.58–0.83) were associated with reduced constipation risk. Furthermore, a high PA was found to decrease constipation risk by 13% when compared with a moderate PA, which was consistent with the results of a previous meta-analysis. These results illustrated that PA serves as an effective means to alleviate symptoms of constipation [44].

### Subgroup analysis of constipation

Based on an analysis of gender and region, the RRs for the protective effect of PA on constipation were not influenced by gender, region, or the number of adjustment factors. Furthermore, we compared the risk estimates considering and not considering adjustment factors to explore whether BMI, gender, age, alcohol consumption, smoking, and fibre intake would affect the relationship between PA and constipation risk. The results indicated that, when adjusting only for BMI, gender, age, alcohol consumption, smoking, and fibre intake, there was no statistically significant inverse relationship between PA and constipation risk. It is important to note that fibre intake is currently considered an important treatment for patients with constipation in primary care, but our study showed it to be a risk factor for constipation; after adjusting for it in our analysis, PA reduced the constipation risk by 42%. One possible explanation is that fibre intake may affect different subtypes of constipation differently [45], and fibre supplementation might be more suitable for specific populations with certain symptoms, leading to increased flatulence [46]. Another possible explanation is the small sample size of the subgroup for fibre intake included in our analysis. Additionally, factors such as BMI, gender, and age are closely associated with constipation and may act as risk factors or impairing factors for its onset. A survey on the constipation prevalence in obese individuals showed that obese people are at a higher risk of experiencing constipation [47]. Other studies have shown relationships between gender and age, and gastrointestinal tract and functional gastrointestinal disorders are more prevalent in women than men. Moreover, ageing is closely related to neural and functional decline in the intestines [48,49]. Basic or cohort studies on the relationship between smoking and drinking and the gastrointestinal tract have shown that alcohol consumption can lead to gastrointestinal motility disorders causing delays in intestinal transit, and smoking increases the risk of various gastrointestinal disorders. Past and current smoking habits are associated with constipation onset [50–52]. Our research results agreed with these findings, demonstrating that adjusting for factors such as BMI, gender, age, alcohol consumption, and smoking showed that PA can reduce constipation risk by 45%, 48%, 40%, 38%, and 40%, respectively.

### Biological mechanisms

Several mechanisms can explain how PA improves constipation. For example, PA can promote intestinal motility, the lack of which is a known cause of constipation, and can prolong colonic transit time and reduces gastrointestinal motility [53]. For individuals with chronic constipation symptoms and accompanying inactivity, regular PA can improve the transit time of the cecum or colon [19]. Furthermore, different types of PA levels can improve gastrointestinal motility, promoting intestinal peristalsis and alleviating constipation symptoms. Aerobic exercise or core strengthening exercise can also reduce colonic transit time [54,55], and high-level PA can significantly reduce total colonic transit time compared with other levels of PA [56], aligning with our observations (i.e. moderate PA and high PA offer more protection against constipation compared with low PA).

An imbalance in the gut microbiota is another potential mechanism of constipation. The gut microbiome can regulate the occurrence of constipation through pathways such as the enteric nervous system, central nervous system, immune system, intestinal secretions, and endocrine hormones [57]. Here, PA can induce qualitative or quantitative changes in the intestinal microbial composition to benefit the body [58]. An animal study demonstrated that PA could alter the intestinal microbiota in rats, increasing the butyrate concentration [59], which is negatively correlated with intestinal transit time, indicating that PA can alleviate constipation symptoms by increasing the butyrate concentration. By contrast, individuals with higher PA levels tend to produce a greater variety of health-related faecal metabolites compared with those who are less active [60]. Therefore, the lack of a significant protective effect of low PA on constipation could be attributed to its limited capacity to enhance microbiota diversity, which is closely linked to more pronounced alleviation of constipation symptoms. Overall, our findings add further support to constipation-related public health guidelines [61,62] that recommend increasing PA in constipated patients.

### Strengths and limitations

The strengths of our study are its robust meta-analysis, supplemented by subgroup analyses based on gender and region, while adjustments were made for smoking, alcohol consumption, age, gender, BMI, and fibre intake to enhance the thoroughness and reliability of the results. In terms of limitations, we should note that several studies included in our analysis did not clearly categorise PA levels, preventing a more precise examination of the relationship between the degree of PA and constipation risk.

### Implications

Our findings emphasised that PA plays a crucial role in initiating early lifestyle changes for treating constipation and offers guidance for managing the daily lives of patients with this condition. Additional research is required to establish the most effective dose, intensity, and PA duration to reduce the risk of constipation and enhance our understanding of its effects, despite the limited research in this area. Future prospective studies should comprehensively investigate the impact of PA on constipation severity by considering all dimensions and domains of PA. In doing so, they should account for all known confounding factors, such as age, sex, smoking, alcohol consumption, BMI, fibre intake, fluid intake, and education.

## CONCLUSIONS

Our comprehensive analysis showed that a moderate-to-high PA level acted as a protective factor against constipation, significantly reducing the overall constipation risk. This supports an inverse relationship between PA and constipation.

## Additional material


Online Supplementary Document
