# Supplementary material for: Physical activity and constipation: A systematic review of cohort studies
Source: J Glob Health. 2024 Nov 22;14:04197. doi: 10.7189/jogh.14.04197 (PMC11583288; doi:10.7189/jogh.14.04197)
Supplement: Online Supplementary Document [file jogh-14-04197-s001.pdf]

Supplementary appendix

Supplement to:

Physical activity and constipation: A systematic review of cohort studies

1. Full search terms

2. Characteristics of the 13 studies on physical activity and constipation risk (Table 1)

3. Quality of studies according to Newcastle-Ottawa Scale (Table 2)

4. Figures

1. Full search strategy

#1 Exercises OR Physical Activity OR Activities, Physical OR Activity, Physical OR Physical Activities OR Exercise, Physical OR Exercises, Physical OR Physical Exercise OR Physical Exercises OR Acute Exercise OR Acute Exercises OR Exercise, Acute OR Exercises, Acute OR Exercise, Isometric OR Exercises, Isometric OR Isometric Exercises OR Isometric Exercise OR Exercise, Aerobic OR Aerobic Exercise OR Aerobic Exercises OR Exercises, Aerobic OR Exercise Training OR Exercise Trainings OR Training, Exercise OR Trainings, Exercise

#2 Constipation OR Dyschezia OR Colonic Inertia

Table S1. Characteristics of the 13 studies on physical activity and constipation risk

Table 1 Characteristics of the 13 studies on physical activity and constipation risk

**Authors,year**	**Gender**	**Region**	**Subjects**	**Cases**	**Relative Risk (95%CI) for high VS low PA**	**Relative Risk (95%CI) for high VS moderate PA**	**Relative Risk (95%CI) for moderate VS low PA**	**Low PA defined by**	**Moderate PA defined by**	**High PA defined by**	**Adjustment factors (excluding)**
Keiko Asakura et al 2017	female and male	Asia	5309	5309	0.60(0.40,0.90)	0.81(0.78,0.83)	0.74(0.49,1.15)	Low	Middle	High	residential area, physical acti- vity level, preparation time f- or breakfast, and educational background of the mother
Gamze Yurtdas et al 2020	female and male	Asia	4561	4561	0.74(0.59,0.90)	0.96(0.95,0.99)	0.77(0.62,0.91)	Inactive	Moderately active	Active	age, gender, BMI, physical a- ctivity level(IPAQ), fiber inta- ke(quartiles), water intake(qua- rtiles)
Shigeyuki Naka ji et al 2002	male	Asia	1699	696	0.46(0.23,1.05)	None	None	≤4h/day	Nnoe	＞4h/day	Nnoe
Migule A Sanjoaquin et al 2004	male	European	20630	4654	1.04(0.69,1.57)	0.97(0.86,1.08)	1.07(0.80,1.45)	None	1-3h/week	7or more h/week	age, diet group, BMI, fibre i- ntake, fluid intake, vigorous exercise, employment status, smoking, alcohol intake and, for women only, menopausal status and current HRT use
	female	European	20630	15976	1.70(1.42,2.03)	1.48(1.37,1.60)	1.15(1.04,1.27)	None	1-3h/week	7or more h/week	age, diet group, BMI fibre in- take, fluid intake, vigorous e-

											xercise, employment status, smoking, alcohol intake and, for women only, menopausal status and current H-RT use
Lisa M Driesse n et al 2013	female and male	European	347	347	0.37(0.15,0.92)	0.84(0.79,0.88)	0.44(0.19,1.04)	Total activity% ＜12	Total activity%12-17	Total activity% ≥17	attendance of childcare and b-irth weight z score. Addition-al adjustment for other covar-iates did to change the effect estimates with ≥ 10%
Ling Huang et al 2017	female	Asia	1568	77	0.61(0.37,0.98)	None	None	≤2 days/ week		≥3 days /week	Nnoe
Parinaz Moezi et al 2018	female and male	Asia	9264	752	0.56(0.46,0.68)	0.76(0.74,0.76)	0.74(0.62,0.89)	Low	Medium	High	Nnoe
Wendy J. Brow n et al 2000	female	Oceania	13609	13609	0.72(0.63,0.83)	0.81(0.79,0.83)	0.89(0.80,1.00)	PA score ＜5	PA score 5-＜15	PA score≥ 25	adjusting for smoking status, alcohol consumption, number of diets in last year, body mass index, lowiron, menopausal status, type of contraception or hormonereplacement therapy, stress level, education, and area of residence.
Rong Huang et al 2014	female and male	Asia	32371	5052	0.79(0.74,0.86)	None	None	＜1hour/ day		≥1hour/ day	adjusting for sex, age, percei-ved family affluence, fruit an d vegetable intake, and depre-ssion and anxiety symptoms

JAMES E Everhart et al 1989	male	North America	14407	4411	0.32(0.14,0.77)	0.56(0.26,1.19)	None	Least	Intermediate	Most	age
	female	North America	14407	6793	0.29(0.20,0.42)	0.56(0.40,0.77)	None	Least	Intermediate	Most	age
Patrick B. Wilson 2020	female and male	North America	9963	771	0.87(0.68,1.12)	0.98(0.74,1.30)	None	Low	Moderate	High	age, gender, education,race/ethnicity, relationship status, perceived health status, BMI, dietary fiber, dietary moisture
Bodil Ohlsson et al 2016	female and male	European	16840	516	0.298(0.153,0.580)	0.552(0.441,0.689)	0.540(0.347,0.842)	Mostly sitting	Activity 30-60min/day	Strenuous activity 60 min/day	basal characteristics
Mari A. Kuutti et al 2023	female	European	1098	189	1.00(0.95,1.04)	0.97(0.68,1.38)	1.03(0.69,1.54)	No exercise	Regular PA	Current PA (MET-h/d)	None

CI - confidence interval % - percent

Table S2. Quality of studies according to Newcastle-Ottawa Scale

Table 2 Quality of studies according to Newcastle-Ottawa Scale

First author,<br>Year, country	Selection<br>(Max, score 4)	Comparability<br>(Max, score 2)	Exposure (case-control) or outcome<br>(cohort) (max, score 3)
Keiko Asakura et al 2017	3	1	2
Gamze Yurtdas et al 2020	3	2	2
Shigeyuki Nakaji et al 2002	2	1	2
Migule A Sanjoaquin et al 2004	3	2	2
Lisa M Driessen et al 2013	2	1	2
Ling Huang et al 2017	4	1	1
Parinaz Moezi et al 2018	3	1	1
Wendy J. Brown et al 2000	2	2	2
Rong Huang et al 2014	4	1	2
JAMES E Everhart et al 1989	2	1	2
Patrick B. Wilson 2020	3	2	2
Bodil Ohlsson 2016	4	2	3
Mari A. Kuutti et al 2023	4	1	2

4. Figures

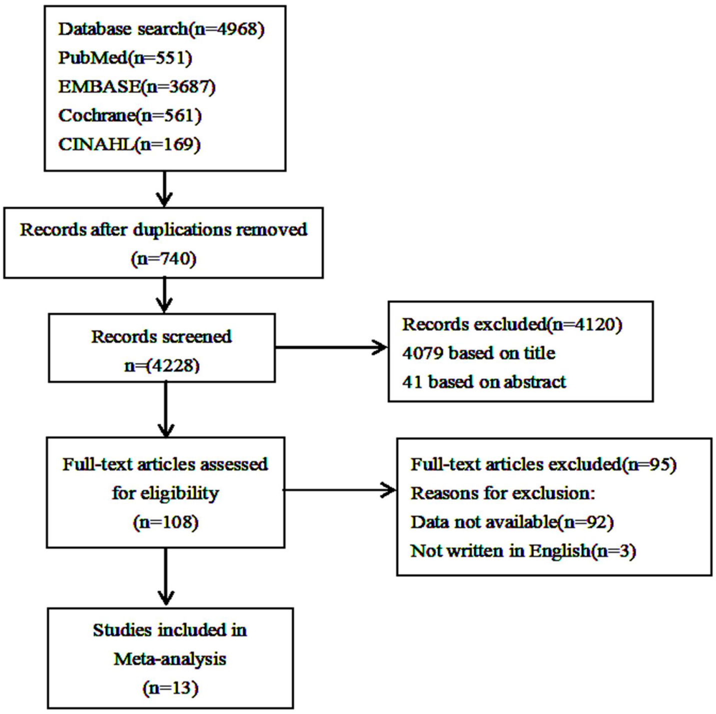

Figure S1. PRISMA fow diagram of identifcation and selection of eligible studies

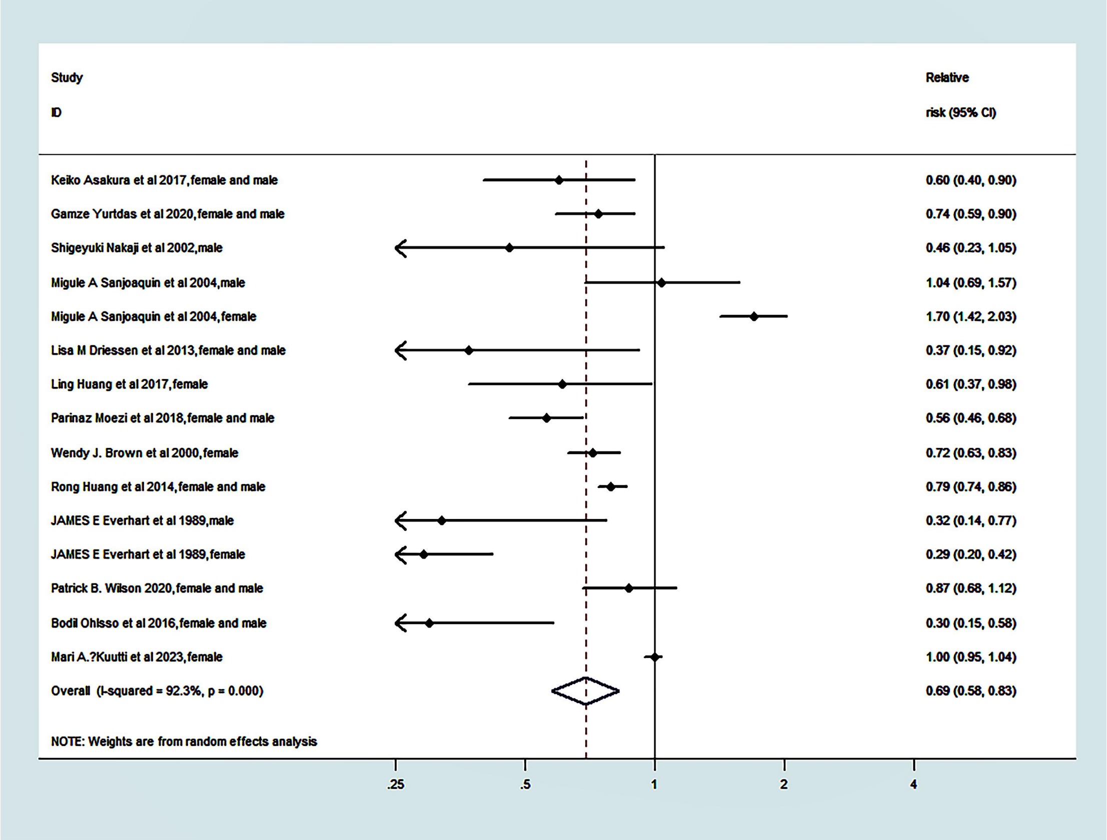

Figure S2. Forest plot of a random efects meta-analysis including 15 risk estimates of constipation for a high versus low level of PA

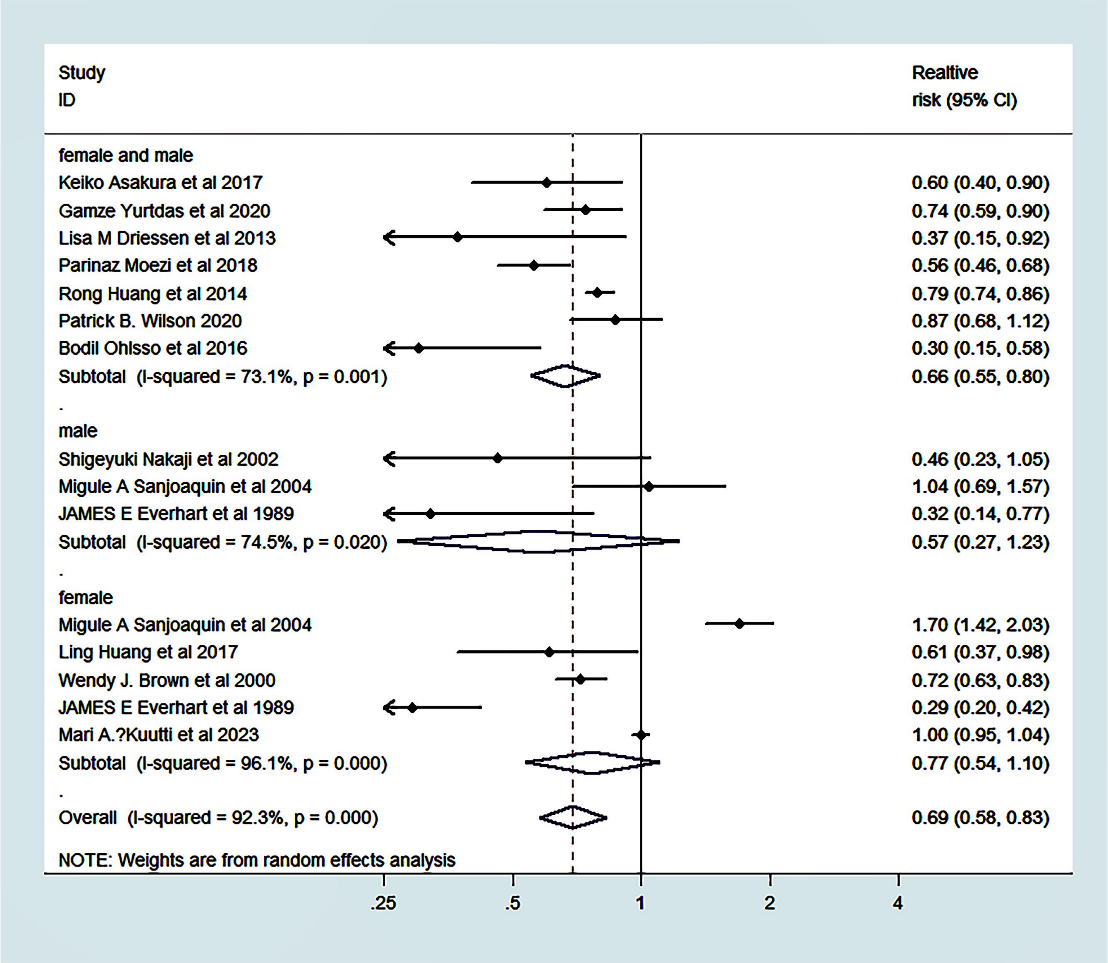

Figure S3. Forest plot of a random efects meta-analysis including 15 risk estimates of constipation for a high versus low level of PA, grouped by gender

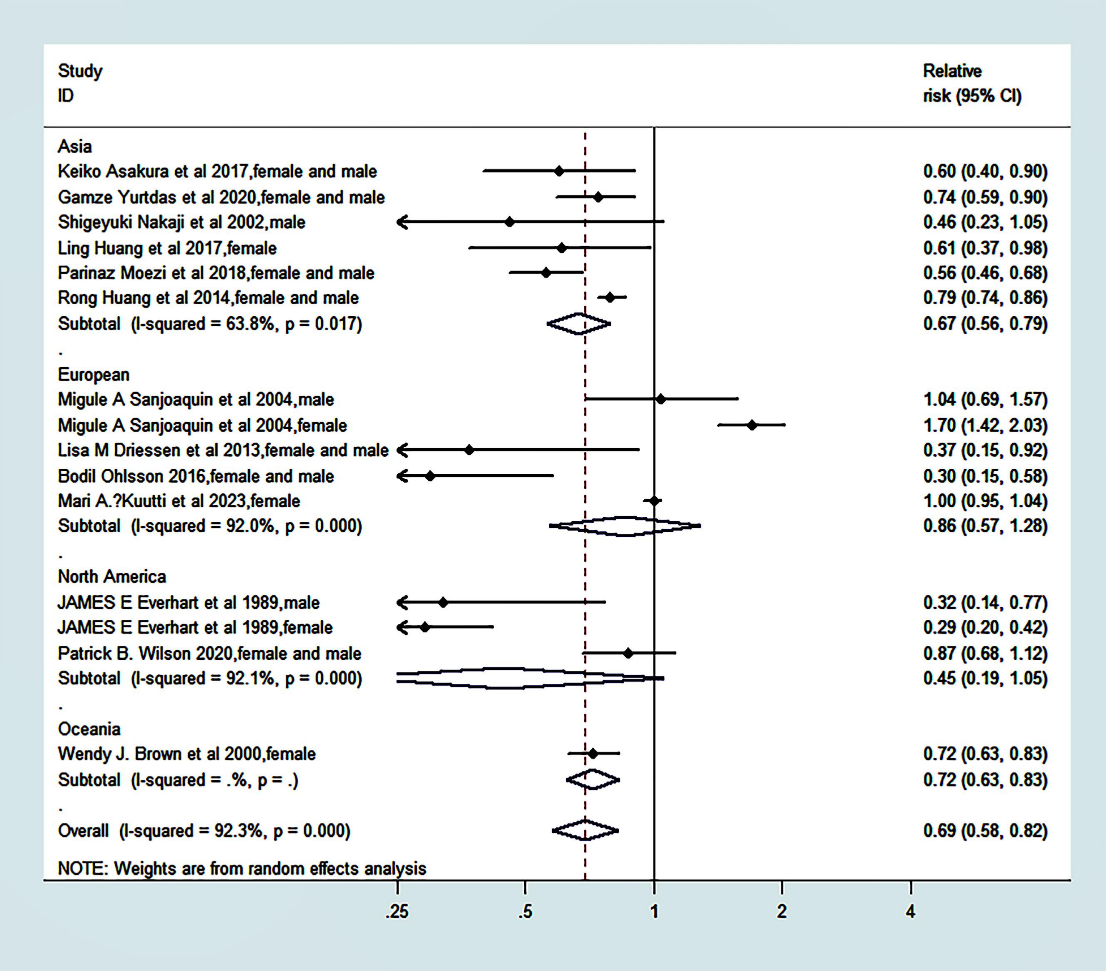

Figure S4. Forest plot of a random efects meta-analysis including 15 risk estimates of constipation for a high versus low level of PA, grouped by region

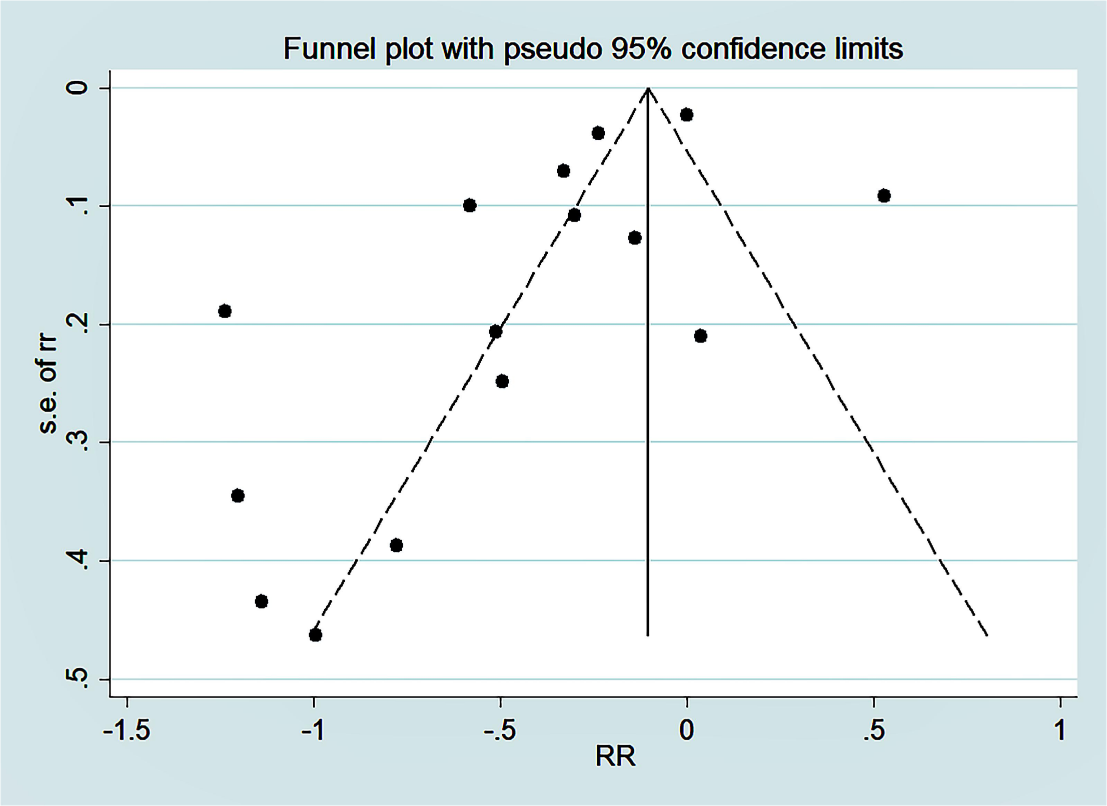

Figure S5. Standardized Funnel plot corresponding to the main random-efects meta-analysis

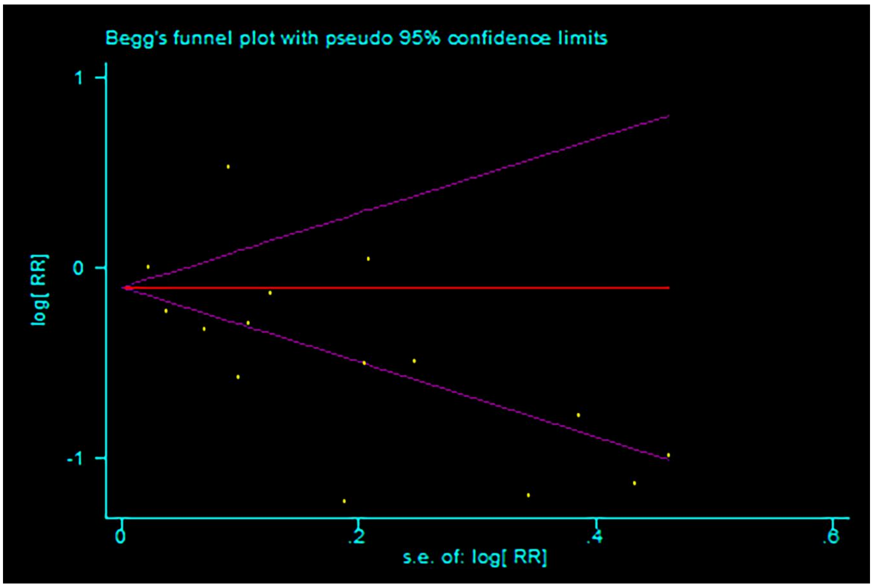

Figure S6. Standardized Begg's rank correlation test corresponding to the main random-efects meta-analysis

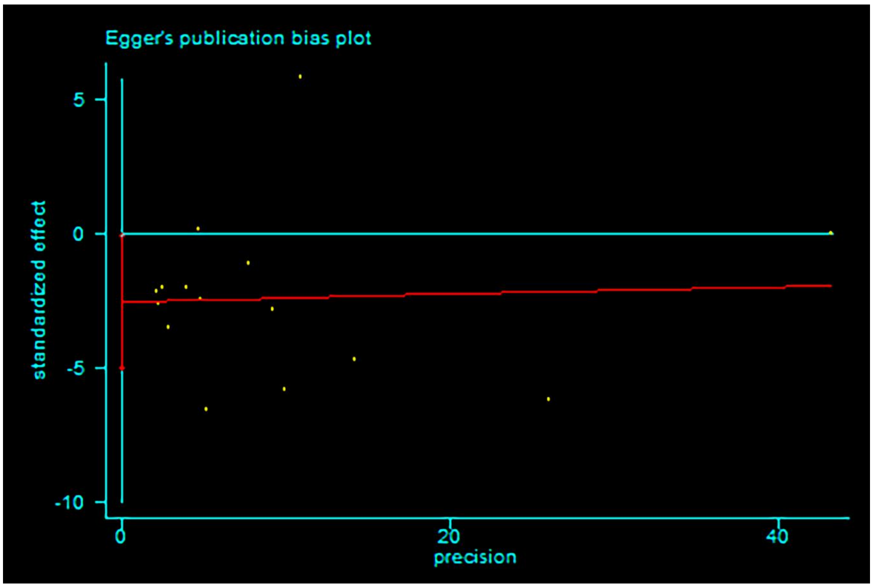

Figure S7. Standardised Egger's regression test corresponding to the main random-efects meta-analysis

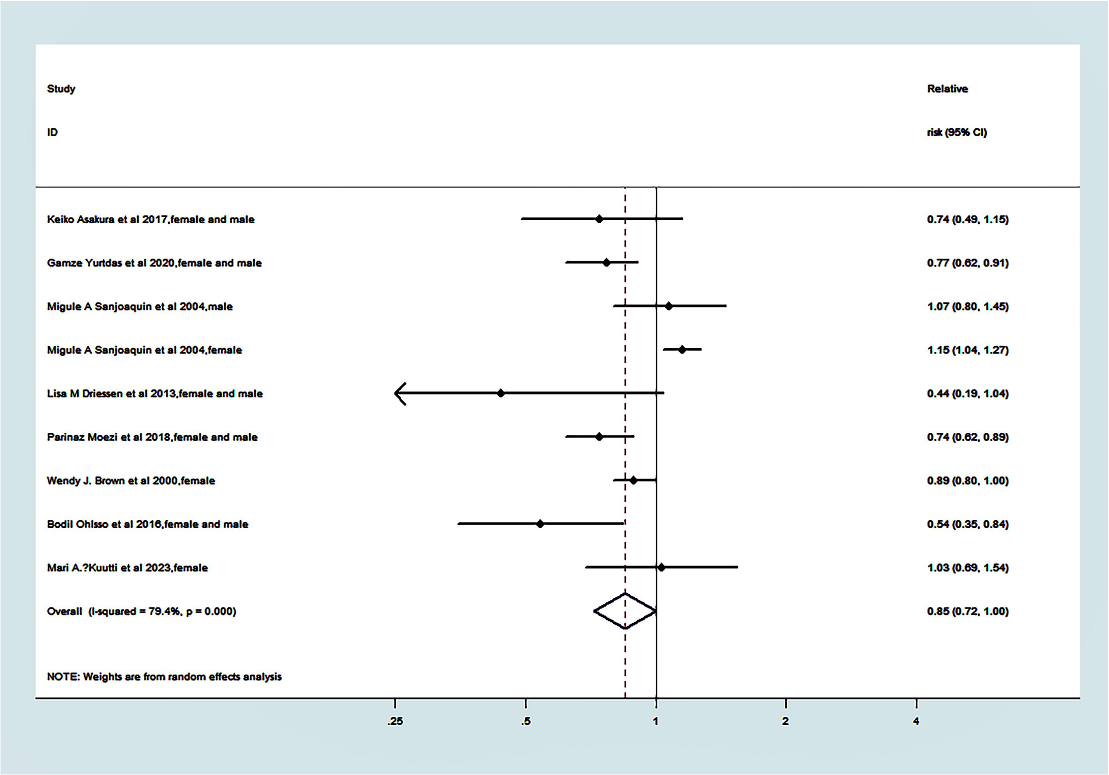

Figure S8. Forest plot of a random efects meta-analysis including 9 risk estimates of constipation for a moderate versus low level of PA

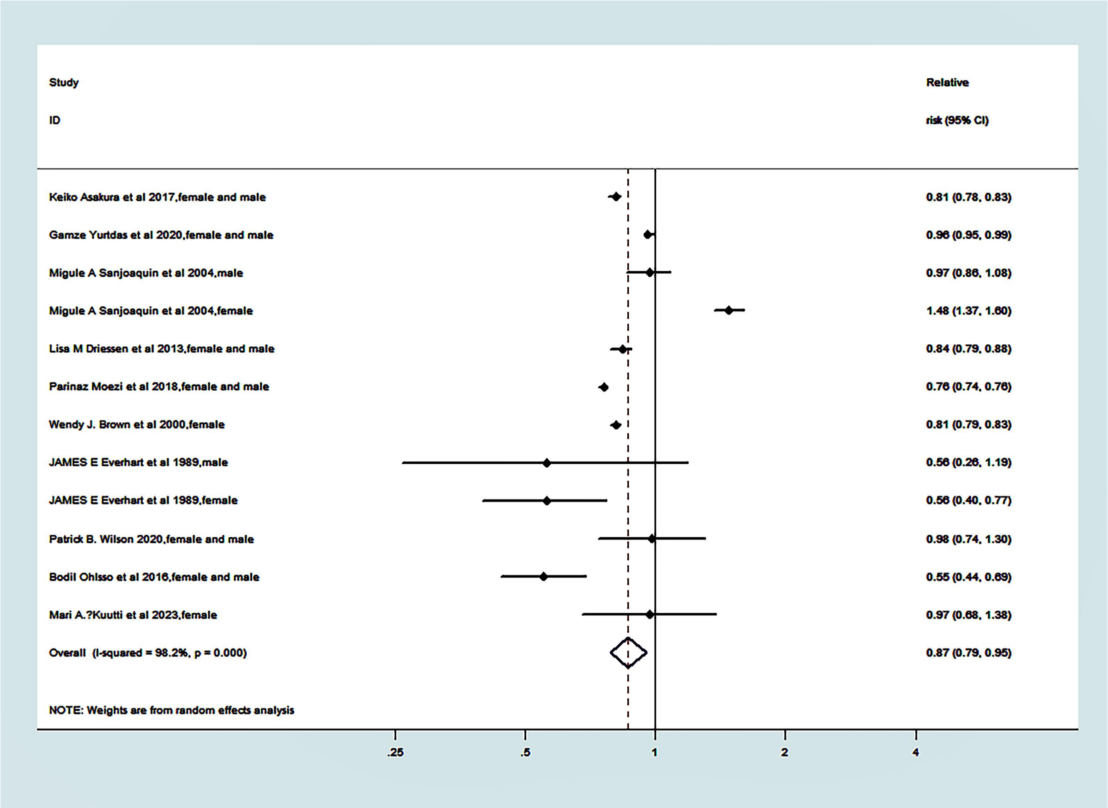

Figure S9. Forest plot of a random efects meta-analysis including 12 risk estimates of constipation for a high versus moderate level of PA

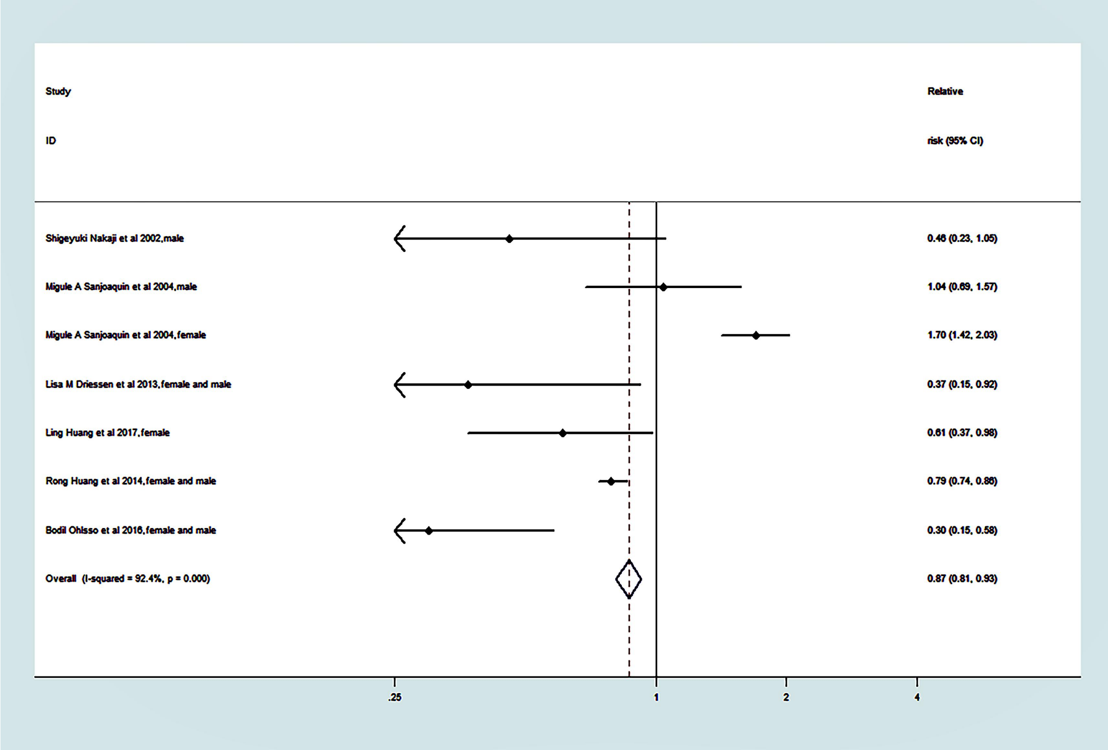

Figure S10. Forest plot of a random efects meta-analysis including 7 risk estimates of constipation for PA guidelines
